# 初诊多发性骨髓瘤患者高危细胞遗传学异常数目对临床特征及预后的影响

**DOI:** 10.3760/cma.j.issn.0253-2727.2022.05.010

**Published:** 2022-05

**Authors:** 智 陈, 园 夏, 睿 郭, 闰 张, 海荣 仇, 媛媛 金, 建勇 李, 丽娟 陈

**Affiliations:** 南京医科大学第一附属医院，江苏省人民医院血液科，南京 210029 Department of Hematology, the First Affiliated Hospital of Nanjing Medical University, Jiangsu Province Hospital, Nanjing 210029, China

**Keywords:** 多发性骨髓瘤, 细胞遗传学异常, 荧光原位免疫杂交, 预后, Multiple myeloma, Cytogenetic abnormalities, Fluorescence in situ hybridization, Prognosis

## Abstract

**目的:**

探讨初诊多发性骨髓瘤（MM）患者的高危细胞遗传学异常（HRCA）数目对临床特征及预后的影响。

**方法:**

选取2013年11月至2020年9月江苏省人民医院收治的360例初诊MM患者，应用胞质轻链免疫荧光结合荧光原位免疫杂交（cIg-FISH）技术检测患者的HRCA，并结合患者的临床资料进行分析。

**结果:**

360例患者中，无HRCA、1种HRCA、2种HRCA、3种HRCA的患者分别为120例（33.3％）、175例（48.6％）、61例（16.9％）、4例（1.1％）。依据HRCA数目将患者分为无HRCA组、1种HRCA组、≥2种HRCA组，三组患者的R-ISS分期、血红蛋白、白蛋白、骨髓浆细胞比例、诱导治疗后疗效差异均有统计学意义（*P*值均<0.05）。Cox比例风险回归分析显示，髓外病变（*P*＝0.018）、HRCA≥2种（*P*＝0.001）、未行自体造血干细胞移植（*P*<0.001）是影响患者无进展生存的独立危险因素，乳酸脱氢酶≥220 U/L（*P*<0.001）、HRCA≥2种（*P*＝0.001）、未行自体造血干细胞移植（*P*＝0.005）是影响患者总生存的独立危险因素。生存分析显示，无HRCA组、1种HRCA组、≥2种HRCA组患者的中位无进展生存期分别为28、22、14个月（*P*＝0.005），中位总生存期分别为未达到、60个月、30个月（*P*＝0.001）。

**结论:**

HRCA数目≥2是影响初诊MM患者生存的独立危险因素，HRCA数目越多，肿瘤负荷越重，进展及死亡风险越高。

## Abstract (en)

**Objective:**

To investigate the influence of the number of high-risk cytogenetic abnormalities（HRCA）on the clinical characteristics and prognosis of patients with newly diagnosed multiple myeloma（MM）.

**Methods:**

A total of 360 patients with newly diagnosed MM admitted to Jiangsu Province Hospital between November 2013 and September 2020 were included in this study. Cytoplasmic light chain immunofluorescence with fluorescence in situ hybridization（cIg-FISH）was used to detect HRCA. Cytogenetic abnormalities were combined with clinical characteristics and outcomes for further analysis.

**Results:**

Among the 360 patients, 120 patients（33.3％）presented with no HRCAs, and 175（48.6％）, 61（16.9％）, and four（1.1％）patients had one, two, and three HRCA（s）, respectively. Patients were divided into three groups, including the no-HRCA group, one-HRCA group, and ≥two-HRCA group, according to the number of HRCAs. There were significant differences in the R-ISS stage, hemoglobin level, albumin level, and the proportion of bone marrow plasma cells among the three groups（*P*<0.05）. The COX proportional-hazards model identified extramedullary disease（*P*＝0.018）, HRCA ≥ 2（*P*＝0.001）, and absence of autologous hematopoietic stem cell transplantation（*P*<0.001）as independent risk factors for progression free survival（PFS）and identified lactate dehydrogenase（LDH）level ≥ 220 U/L（*P*<0.001）, HRCA ≥2（*P*＝0.001）, and absence of autologous hematopoietic stem cell transplantation（*P*＝0.005）as independent risk factors for overall survival（OS）. The median PFS was 28 months, 22 months, and 14 months（*P*＝0.005）for the three cohorts, and their OS was not reached, 60 months, and 30 months（*P*＝0.001）, respectively.

**Conclusion:**

HRCA ≥ 2 is an independent risk factor for decreased survival in patients with newly diagnosed MM. More HRCAs result in heavier tumor burden, as well as a higher risk of disease progression and death.

多发性骨髓瘤（MM）是常见的浆细胞恶性疾病，目前仍被认为是一种不可治愈的疾病[1]。细胞遗传学异常是影响MM患者预后的重要因素之一，多数MM患者都伴有不同程度的细胞遗传学异常。目前应用最为广泛的修订的国际分期系统（R-ISS）将del（17p）、t（4;14）、t（14:16）定义为高危细胞遗传学异常（high-risk cytogenetic abnormality, HRCA）[2]；Mayo骨髓瘤分层及风险调整治疗（Mayo Stratification of Myeloma and Risk-Adapted Therapy, mSMART）共识在此基础上增加gain（1q21）、t（14;20）为HRCA，并指出这5种HRCA中出现2种及以上为预后很差的“双打击”骨髓瘤[3]；另有研究指出TP53（位于染色体17p13）双等位基因失活，或者出现国际分期系统（ISS）Ⅲ期合并CKS1B（位于染色体1q21）扩增提示预后极差[4]。由于目前如何通过细胞遗传学定义高危MM仍存在一定争议，且对MM患者HRCA的大宗研究主要来自欧美国家，因此，本研究通过胞质轻链免疫荧光结合荧光原位免疫杂交（cytoplasmic light chain immunofluorescence with fluorescence in situ hybridization, cIg-FISH）技术检测了360例初诊MM患者的细胞遗传学异常，拟通过分析患者HRCA数目对临床特征及预后的影响进一步探讨HRCA在中国MM患者中的意义。

## 病例与方法

1. 病例：选取2013年11月至2020年9月江苏省人民医院血液科收治的初诊MM患者360例，MM的诊断标准符合《中国多发性骨髓瘤诊治指南（2020年修订）》[5]。本研究经江苏省人民医院医学研究伦理委员会批准（No.2020-SR-589），并获得患者知情同意。本研究中对髓外病变的定义包括骨相关髓外病变和骨外髓外病变。

2. cIg-FISH的检测和判定：所有患者均在初诊时通过cIg-FISH技术检测细胞遗传学改变。由于仅有部分IgH易位伙伴基因不明确的患者进行了t（14;20）检测，且根据既往报道，t（14;20）阳性率不足1％[3]，因此本研究未纳入t（14;20）的结果。采用我们前期研究中使用的cIg-FISH检测方法[6]，将骨髓标本依次进行单个核细胞分离、制片、固定、烤片、变性、脱水后，与相应的探针进行杂交，随后洗片、胞质轻链染色、封片，在荧光显微镜下计数100个浆细胞进行判断。采用美国Abbott公司探针进行gain/amp（1q21）、del（17p）、14q32断裂、t（4;14）、t（11;14）、t（14;16）的检测。参考国内外标准[7]–[8]，将上述检测的阳性阈值分别定为：gain/amp（1q21）和del（17p）为20％，IgH（14q32）断裂及易位为10％。参照mSMART共识，将del（17p）、gain（1q21）、t（4;14）、t（14:16）定义为HRCA[3]。

3. 随访：通过查阅患者住院或门诊病历以及电话方式对所有患者进行随访，随访截止时间为2021年1月，中位随访时间25（1～82）个月。

4. 统计学处理：应用SPSS 20.0及Graphpad Prism 7.0软件进行统计学分析。计数资料的比较采用*χ*^2^检验；预后影响因素的分析采用Cox比例风险回归模型；生存分析采用Kaplan-Meier曲线，生存的比较采用Log-rank法。全部统计学方法使用双侧检验，*P*<0.05为差异有统计学意义。

## 结果

1. 初诊MM患者HRCA的发生率：360例患者中，男197例、女163例，中位年龄62（26～90）岁。199例（55.3％）患者出现了1q21重复，其中gain（1q21）即1q21拷贝数为3的患者118例（32.8％），amp（1q21）即1q21拷贝数≥4的患者81例（22.5％）；del（17p）阳性患者39例（10.8％）；t（4;14）阳性患者67例（18.6％）；t（14:16）阳性患者4例（1.1％）。

对所有患者的HRCA数目进行统计，无HRCA的患者120例（33.3％），1种HRCA的患者175例（48.6％），2种HRCA的患者61例（16.9％），3种HRCA的患者4例（1.1％）。

2. 不同HRCA数目患者的临床特征、治疗方案及疗效的比较：依据HRCA数目将所有患者分为三组：无HRCA组、1种HRCA组、≥2种HRCA组。对患者初诊时临床特征的比较显示，三组患者在R-ISS分期、血红蛋白、白蛋白、骨髓浆细胞比例的比较中差异均有统计学意义（*P*值均<0.05），而在性别、年龄、DS分期、ISS分期、乳酸脱氢酶、肌酐、血清钙、β_2_微球蛋白、有无髓外病变方面差异均无统计学意义（*P*值均>0.05）。对患者治疗方法的比较显示，三组患者在诱导治疗中使用以硼替佐米和（或）来那度胺为基础的方案的比例差异有统计学意义（*P*＝0.002），而进行自体造血干细胞移植的比例差异无统计学意义（*P*>0.05）。对患者诱导治疗后疗效的比较显示，三组患者≥非常好的部分缓解（VGPR）的比例差异有统计学意义（*P*＝0.023）（表1）。

**表1:** 不同高危细胞遗传学异常（HRCA）数目的初诊多发性骨髓瘤患者临床特征、治疗及疗效的比较［例（％）］

特征	无HRCA组（120例）	1种HRCA组（175例）	≥2种HRCA组（65例）	*P*值
性别				0.904
男	66(55.0)	94(53.7)	37(56.9)	
女	54(45.0)	81(46.3)	28(43.1)	
年龄（岁）				0.176
<65	73(60.8)	112(64.0)	33(50.8)	
≥65	47(39.2)	63(36.0)	32(49.2)	
DS分期				0.887
Ⅰ~Ⅱ	21(17.5)	27(15.4)	11(16.9)	
Ⅲ	99(82.5)	148(84.6)	54(83.1)	
ISS分期				0.484
Ⅰ~Ⅱ	59(49.2)	79(45.1)	26(40.0)	
Ⅲ	61(50.8)	96(54.9)	39(60.0)	
R-ISS分期				<0.001
Ⅰ~Ⅱ	110(91.7)	134(76.6)	28(43.1)	
Ⅲ	10(8.3)	41(23.4)	37(56.9)	
血红蛋白				0.001
<100 g/L	71(59.2)	136(77.7)	50(76.9)	
≥100 g/L	49(40.8)	39(22.3)	15(23.1)	
白蛋白				0.040
<35 g/L	72(60.0)	116(66.3)	51(78.5)	
≥35 g/L	48(40.0)	59(33.7)	14(21.5)	
乳酸脱氢酶				0.660
<220 U/L	107(89.2)	154(88.0)	55(84.6)	
≥220 U/L	13(10.8)	21(12.0)	10(15.4)	
肌酐				0.454
<177 µmol/L	88(73.3)	134(76.6)	53(81.5)	
≥177 µmol/L	32(26.7)	41(23.4)	12(18.5)	
血清钙				0.402
≤2.65 mmol/L	96(80.0)	143(81.7)	48(73.8)	
>2.65 mmol/L	24(20.0)	32(18.3)	17(26.2)	
β_2_微球蛋白				0.926
<5.5 mg/L	59(49.2)	84(48.0)	30(46.2)	
≥5.5 mg/L	61(50.8)	91(52.0)	35(53.8)	
骨髓浆细胞比例				0.045
<30％	79(65.8)	94(53.7)	32(49.2)	
≥30％	41(34.2)	81(46.3)	33(50.8)	
有无髓外病变				0.537
无	99(82.5)	143(81.7)	57(87.7)	
有	21(17.5)	32(18.3)	8(12.3)	
诱导治疗方案				0.002
硼替佐米和（或）来那度胺为基础的方案	90(75.0)	153(87.4)	60(92.3)	
传统化疗方案	30(25.0)	22(12.6)	5(7.7)	
是否行ASCT				0.296
是	16(13.3)	35(20.0)	13(20.0)	
否	104(86.7)	140(80.0)	52(80.0)	
诱导治疗后疗效				0.023
<VGPR	45(37.5)	77(44.0)	38(58.5)	
≥VGPR	75(62.5)	98(56.0)	27(41.5)	

注：ASCT：自体造血干细胞移植；VGPR：非常好的部分缓解

3. 初诊MM预后影响因素的分析：采用Cox比例风险回归模型分析患者预后的影响因素，在单因素回归分析之后，将*P*<0.05的变量进一步纳入多因素回归分析。对无进展生存（PFS）期的分析显示，髓外病变（*P*＝0.018）、HRCA≥2种（*P*＝0.001）是影响患者PFS的独立危险因素，自体造血干细胞移植（*P*<0.001）是影响患者PFS的独立保护因素（表2）。对总生存（OS）期的分析显示，乳酸脱氢酶≥220 U/L（*P*<0.001）、HRCA≥2种（*P*＝0.001）是影响患者OS的独立危险因素，自体造血干细胞移植（*P*＝0.005）是影响患者OS的独立保护因素（表2）。

**表2:** 影响多发性骨髓瘤患者生存的单因素和多因素回归分析

影响因素	无进展生存				总生存			
	单因素分析		多因素分析		单因素分析		多因素分析	
	*HR*(95％ *CI*)	*P*值	*HR*(95％ *CI*)	*P*值	*HR*(95％ *CI*)	*P*值	*HR*(95％ *CI*)	*P*值
男性	1.04(0.78~1.38)	0.805			1.18(0.80~1.75)	0.408		
年龄≥65岁	1.27(0.94~1.70)	0.115			1.74(1.18~2.58)	0.005	1.21(0.80~1.82)	0.372
DS分期Ⅲ级	1.17(0.79~1.73)	0.428			0.86(0.52~1.42)	0.556		
ISS分期Ⅲ级	1.36(1.01~1.82)	0.040	0.88(0.48~1.61)	0.675	1.87(1.24~2.81)	0.003	1.08(0.50~2.34)	0.854
R-ISS分期Ⅲ级	1.21(0.88~1.68)	0.242			1.47(0.97~2.24)	0.072		
髓外病变	1.45(1.02~2.05)	0.038	1.54(1.08~2.19)	0.018	1.41(0.89~2.23)	0.138		
骨髓浆细胞比例≥30％	1.11(0.83~1.48)	0.484			1.06(0.72~1.57)	0.769		
血红蛋白<100 g/L	0.93(0.68~1.28)	0.675			1.04(0.67~1.59)	0.874		
白蛋白<35 g/L	0.80(0.59~1.07)	0.131			0.92(0.61~1.37)	0.668		
肌酐≥177 µmol/L	1.37(0.99~1.90)	0.055			1.57(1.03~2.39)	0.036	1.14(0.70~1.86)	0.594
血清钙>2.65 mmol/L	1.43(1.03~2.01)	0.035	1.32(0.93~1.88)	0.126	1.85(1.21~2.82)	0.005	1.54(0.98~2.43)	0.062
乳酸脱氢酶≥220 U/L	1.82(1.22~2.72)	0.004	1.50(1.00~2.26)	0.053	3.14(1.95~5.07)	<0.001	2.64(1.62~4.31)	<0.001
β_2_微球蛋白≥5.5 mg/L	1.39(1.04~1.86)	0.026	1.33(0.73~2.40)	0.352	1.85(1.23~2.76)	0.003	1.25(0.59~2.65)	0.556
HRCA≥2种	1.34(1.09~1.65)	0.006	1.42(1.15~1.74)	0.001	1.62(1.22~2.14)	0.001	1.61(1.22~2.15)	0.001
以硼替佐米和（或）来那度胺为基础的诱导治疗方案	0.92(0.68~1.24)	0.573			0.96(0.64~1.43)	0.837		
自体造血干细胞移植	0.32(0.19~0.54)	<0.001	0.34(0.20~0.56)	<0.001	0.22(0.09~0.54)	0.001	0.27(0.11~0.67)	0.005

注：HRCA：高危细胞遗传学异常

4. HRCA数目对患者生存的影响：生存分析结果显示，无HRCA组、1种HRCA组、≥2种HRCA组的中位PFS期分别为28、22、14个月，三组患者PFS的差异有统计学意义（*P*＝0.005）（图1A）；无HRCA组、1种HRCA组、≥2种HRCA组的中位OS期分别为未达到、60个月、30个月，三组患者OS的差异有统计学意义（*P*＝0.001）（图1B）。生存分析结果表明HRCA数目越多，患者预后更差。

**图1:**
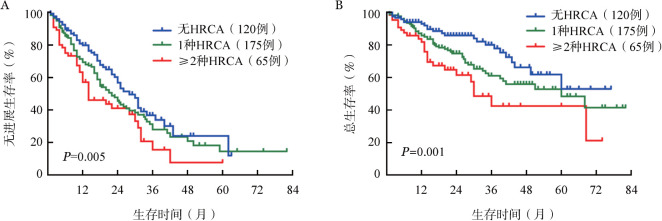
高危细胞遗传学异常（HRCA）数目对初诊多发性骨髓瘤患者预后的影响 A：不同HRCA数目患者的无进展生存比较；B：不同HRCA数目患者的总生存比较

## 讨论

MM是起源于浆细胞的恶性肿瘤，发病率高居血液系统肿瘤第二位。尽管随着新药的广泛应用以及自体造血干细胞移植、CAR-T免疫治疗等技术的不断发展，MM患者的预后得到了极大的改善，但仍有部分患者疗效差、预后不良[1]，因此，早期准确识别这类患者并制定相应的治疗策略具有重要的临床现实意义。

绝大多数MM患者都伴有不同程度的细胞遗传学异常，由于细胞遗传学改变可影响肿瘤细胞的生物学特征，因此被认为是决定MM预后最重要的因素之一[9]。随着MM的治疗手段及遗传学检测方法的不断更新，对HRCA的认识也在不断发展，且在不同的研究中对HRCA的定义不完全相同。2015年国际骨髓瘤工作组在先前制定的ISS的基础上纳入了细胞遗传学的改变，将del（17p）、t（4;14）、t（14;16）定义为HRCA，据此修订形成的R-ISS可以更好地对患者预后进行分层[10]。纳入了1069例初诊MM患者的MRC Myeloma Ⅸ研究根据生存分析将gain（1q21）、del（17p）、t（4;14）、t（14;16）、t（14;20）这5种异常定义为HRCA，并发现携带≥2种HRCA的患者中位PFS期和OS期仅分别为11.7、21.7个月，明显短于携带更少的HRCA的患者[11]。随后的一项研究在MRC Myeloma Ⅸ研究的基础上加入了NCRI Myeloma Ⅺ研究的结果，共纳入了1905例初诊MM患者，并提出前述5种HRCA中出现2种为“双打击（double-hit, DH）”骨髓瘤，出现3种及以上则为“三打击（triple-hit, TH）”骨髓瘤，该研究进一步佐证了基于这5种HRCA定义的DH/TH预示的高进展及死亡风险[12]。2018年美国梅奥医学中心在对mSMART的更新中加入了DH/TH的概念以强调其对预后的重要性[13]。2019年Walker等[4]基于二代测序结合生存分析提出了不同的DH定义，即出现双等位基因TP53失活，或者出现ISS分期Ⅲ期合并CKS1B扩增，这类患者的中位PFS期为15.4个月，中位OS期仅20.7 个月，相较于FISH技术，二代测序从更精准的角度再次证实了del（17p）和gain（1q21）对预后的影响。

目前关于MM中HRCA的大型研究结果主要来自于欧美人群，随着我国人口结构、生活方式等诸多方面的变化，MM发病率逐年升高[14]，因此对中国MM人群进行深入研究具有重要的临床现实意义。一项国内的研究比较了96例初诊MM患者的生存数据发现，在携带1种HRCA的基础上同时出现其他类型的细胞遗传学异常（无论是否为高危类型）可进一步影响患者的生存[15]。另一项国内多中心研究纳入了5个中心的共1015例初诊MM患者，根据mSMART的分层标准，454例患者具备完整的HRCA评估资料，其中携带1种、2种、≥3种HRCA的患者分别占40.5％、14.3％、2.9％，我中心结果与之相近；遗憾的是该研究未进一步分析中国人群中不同HRCA数目患者的预后[16]。

本研究共纳入了360例初诊MM患者，并按照HRCA数目对患者进行分组，比较了不同HRCA数目患者的临床特征、治疗方法及预后。临床特征比较结果显示，不同HRCA数目患者的血红蛋白、白蛋白、骨髓浆细胞比例之间差异有统计学意义，提示HRCA数目越多可能预示着更高的肿瘤负荷。对患者治疗方法的比较发现，HRCA数目越多，使用基于硼替佐米和（或）来那度胺等新药的诱导方案和应用自体造血干细胞移植的比例越高，由于本研究为回顾性研究，因此在不同危险分层患者的治疗选择中存在一定的偏倚。尽管HRCA数目越多的患者更多地应用了新药及自体造血干细胞移植，但这些患者在诱导治疗后达到VGPR及以上的比例更低，生存分析显示这些患者的PFS期及OS期更短，显示了HRCA对不良预后的预测作用，这也在我们的Cox回归分析中得到了进一步验证，提示HRCA≥2种是影响MM患者生存的独立危险因素。我中心结果显示，携带≥2种HRCA患者的中位PFS期及OS期分别为14个月、30个月，PFS与国内外研究基本相近，OS与国内的一项研究结果类似[15]，但较前文所述的国外研究的OS期更长[11]，可能与该研究时间跨度较长因而早期入组的患者未能普遍应用蛋白酶体抑制剂、新型免疫调节剂、自体造血干细胞移植等有关，也可能与人种及地区差异有关。

在这5种HRCA中，染色体1q21重复在初诊MM患者中发生率最高，约35％[17]。1q拷贝数的异常可引起CKS1B基因的上调，进而引起染色体结构不稳定[18]，还可累及PSMD4、MCL1、ANP32E、IL6R等基因，导致疾病的发生、进展及耐药[19]。gain（1q21）是指患者的染色体1q21出现3个拷贝，这类患者预后较差，中位PFS期约2年、中位OS期约5年[3]；国外研究显示，15％的MM患者可出现amp（1q21）（1q21≥4个拷贝），这类患者与gain（1q21）相比预后更差[20]。多项国内研究结果显示，gain（1q21）阳性率为25.5％～28.8％、amp（1q21）阳性率为13.8％～15.9％，且gain（1q21）与amp（1q21）患者的生存未见明显差异[21]–[23]，提示不同人种gain/amp（1q21）的发生率及预后均存在一定差异。本中心gain（1q21）、amp（1q21）发生率分别为32.8％、22.5％，gain（1q21）检出率与国内其他中心相近，amp（1q21）略高于其他中心，可能与不同地区人口及各中心检测方法差异有一定关系。

TP53是位于染色体17p13的抑癌基因，del（17p）可影响TP53的生物学作用而使其抑癌活性丧失，导致复杂的遗传学异常，形成基因组不稳定[24]。del（17p）在MM中的发生率为7％～13％[17],[25]，这些患者预后较差，具有更高的早期进展和死亡风险[2]。本研究中del（17p）的检出率为10.8％，与国内外报道的发生率相近；本中心既往研究同样显示其为不良预后因素，且硼替佐米治疗不能改善其带来的生存劣势[6]。

半数以上的MM患者可出现染色体14q32上的免疫球蛋白重链（IgH）基因的易位，该基因的易位常被认为是起始遗传学改变，即驱动MM发生的因素之一[26]。IgH与4p16（MMSET/FGFR3）、16q23（MAF）、20q11（MAFB）易位分别形成的t（4;14）、t（14;16）、t（14;20）是MM中的HRCA，这些与IgH发生易位的癌基因可能受到IgH增强子的调控而表达失调，进而促使浆细胞永生化的形成，并可在疾病进程中促进细胞增殖、迁移、耐药的发生，最终导致不良预后[3],[26]。

综上所述，本研究共纳入了360例初诊MM患者，是国内较大的单中心MM患者遗传学特征研究。对不同HRCA数目患者的临床特征的分析发现，HRCA数目≥2是初诊MM患者的独立危险因素，HRCA数目越多，肿瘤负荷越高、患者预后越差。尽管部分细胞遗传学异常的发生率在种族之间存在些许差异，mSMART中的HRCA指标仍可精确识别中国初诊MM人群中的高危患者。对于这类高危、尤其是超高危患者的治疗，有待于国内更多的前瞻性、多中心的研究，为中国MM患者的精准治疗提供更多依据。
